# The membrane-tethered transcription factor ANAC089 serves as redox-dependent suppressor of stromal ascorbate peroxidase gene expression

**DOI:** 10.3389/fpls.2012.00247

**Published:** 2012-11-09

**Authors:** Peter Klein, Thorsten Seidel, Benedikt Stöcker, Karl-Josef Dietz

**Affiliations:** Biochemistry and Physiology of Plants, W5-134, Faculty of Biology and CeBiTec, Bielefeld UniversityBielefeld, Germany

**Keywords:** ascorbate peroxidase, gene expression, redox regulation, retrograde signaling, transcription factor

## Abstract

The stromal ascorbate peroxidase (sAPX) functions as central element of the chloroplast antioxidant defense system. Its expression is under retrograde control of chloroplast signals including redox- and reactive oxygen species-linked cues. The sAPX promoter of *Arabidopsis thaliana* was dissected in transient reporter assays using mesophyll protoplasts. The study revealed regulatory elements up to –1868 upstream of the start codon. By yeast-one-hybrid screening, the transcription factor ANAC089 was identified to bind to the promoter fragment 2 (–1262 to –1646 bp upstream of translational initiation). Upon mutation of the *cis*-acting element CACG, binding of ANAC089 was abolished. Expression of a fused fluorescent protein version and comparison with known endomembrane markers localized ANAC089 to the *trans***-**Golgi network and the ER. The transcription factor was released upon treatment with reducing agents and targeted to the nucleus. Transactivation assays using wild type and mutated versions of the promoter showed a partial suppression of reporter expression. The data indicate that ANAC089 functions in a negative retrograde loop, lowering sAPX expression if the cell encounters a highly reducing condition. This conclusion was supported by reciprocal transcript accumulation of ANAC089 and sAPX during acclimation to low, normal, and high light conditions.

## INTRODUCTION

Photosynthetic metabolism involves light driven electron transfer reactions, production of metabolic intermediates with high reducing potential and the generation of reactive oxygen species (ROS). The photosynthesizing chloroplast is equipped with an intricate redox sensory system, a multilayered antioxidant defense and diverse repair mechanisms to minimize ROS-dependent damage. On the one hand, appropriate sensing allows for using redox and ROS information to adjust photosynthetic metabolism and tune gene expression in the plastids but also in the nuclear genome by retrograde signal transfer from the chloroplast to the nucleus (Baier and Dietz, 2005). Pogson et al. (2008) distinguished retrograde signaling in developmental and operational control. Operational control coordinates nuclear gene expression with the actual needs of photosynthetic metabolism. Analyses of transcriptional dynamics in response to environmental or pharmacological perturbations and elaborated mutant screenings have allowed researchers to pinpoint to signaling cues triggering retrograde signaling in operational control (Leister, 2012). Accumulation of singlet oxygen, changes in redox state of intersystem electron transport chain, redox state of metabolites or proteins as for example thioredoxin downstream of photosystem I, intermediates of chlorophyll synthesis and hormone precursors such as for abscisic acid are linked to specific changes in transcript abundance of nuclear encoded chloroplast proteins (Pfannschmidt, 2010). While ROS generation in the illuminated chloroplast is mostly governed by primary light reactions and counteracted by mechanisms that quench excess excitation energy (Li et al., 2009), ROS are decomposed by high capacity antioxidant systems (Asada, 1999; Dietz et al., 2006).

Superoxide is dismutated by CuZn- and Fe-SOD, and hydrogen peroxide decomposed by thylakoid-bound and stromal ascorbate peroxidase (tAPX, sAPX; Nakano and Asada, 1981) or thiol-dependent peroxidases (Dietz, 2011). It has been estimated that thiol-dependent peroxidase activity reaches about 40% and ascorbate-dependent activity about 60% of total hydrogen peroxide decomposition activity in chloroplasts (Dietz et al., 2006). Both systems are prone to inhibition and thus are subjected to turnover, sAPX and tAPX in particular if ascorbate concentrations are low (Miyake and Asada, 1996). Their expression in the nucleus must be under retrograde control to cope with demand for antioxidant capacity (Oelze et al., 2012).

sAPX and tAPX are encoded by separate nuclear genes in *Arabidopsis thaliana*. Complete deletion of sAPX and tAPX is compensated in *A. thaliana* under regular growth conditions (Kangasjärvi et al., 2008). Symptoms of oxidative stress in these plants develop in stressful environment and in particular during early seedling development. *sAPX* and *tAPX* transcript levels are regulated in dependence on developmental state of the leaves (Pena-Ahumada et al., 2006) and environmental cues, such as light intensity (Oelze et al., 2012). After transfer of low or normal light acclimated *A. thaliana* to 100- or 10-fold higher light intensity, respectively, *sAPX*-mRNA levels start to increase after 30 min and reach a maximum at 3–6 h after transfer to high light (Oelze et al., 2012). In contrast to transcript regulation, sAPX and tAPX protein levels respond much less.

Signaling pathways often innervate transcription factors that modulate target gene expression in response to environmental stimuli (Golldack et al., 2011). However despite the importance of chloroplast ascorbate peroxidases in antioxidant defense, transcription factors involved in their regulation have not been described. Based on transcript regulation it can be assumed, that redox and ROS signals might be involved in regulating *tapx* and *sapx* gene expression. Transcriptome analyses have differentiated O_2_^.–^/H_2_O_2_-dependent regulation from singlet oxygen-dependent regulation (op den Camp et al., 2003). *sAPX* was not among the significantly regulated transcripts responding to methylviologen or to illumination of protochlorophyllide accumulating *flu* mutants (op den Camp et al., 2003). Compensatory retrograde regulation is apparent from knock down lines of *A. thaliana* deficient in the alternative H_2_O_2_-detoxifying 2-Cys peroxiredoxin (Baier et al., 2000) where *sAPX* and *tAPX* transcripts were up-regulated, and in double knock out of tAPX and sAPX (Kangasjärvi et al., 2008) where 2-Cys peroxiredoxin protein levels were increased. Apparently there is a delicate feedback from the chloroplast antioxidant defense system to nuclear gene expression.

The aim of the study was to approach a better and mechanistic understanding of *sapx* gene expression regulation. The promoter was analyzed for regulatory regions. The transcription factor ANAC089 identified in a yeast-one-hybrid (Y1H) screening was subjected to a more detailed inspection for binding site and regulatory properties. The specific promoter region *sapx2*-1 proved to be responsive to oxidative versus reductive cues by ANAC089. The obtained data indicate a role of ANAC089 as repressor of sapx gene activity under highly reducing conditions where the need for sAPX expression or sAPX turnover might be low.

## MATERIALS AND METHODS

### PLANT GROWTH AND LIGHT TREATMENT

*Arabidopsis thaliana* was grown in a growth chamber at a photoperiod of 10 h with 80 µmol quanta m^–2^ s^–1^ and 21°C, and a dark phase of 14 h at 18°C, both at 50% relative humidity. The pots contained Spezialsubstrat (Stender AG, Schermbeck, Germany), Osmocote Start as fertilizer (Scotts Australia PTY Ltd, Bella Vista, Australia), and one tablet of Lizetan (Bayer, Leverkusen, Germany) per L soil. Three week old plants were either transferred to 8 µmol quanta m^–2^ s^–1^ (low light, L-light) or kept at 80 µmol quanta m^–2^ s^–1^ (normal light, N-light) for another 10 days. Then the 4.5 week old plants were exposed to high light (H-light, 800 µmol quanta m^–2^ s^–1^) 1 h after onset of light (9 am). Control plants were kept in L- or N-light. At 3 p.m. complete rosettes of 4–12 plants from the four treatments were harvested, frozen in liquid nitrogen, and stored at –80°C until further processing.

### TRANSCRIPT ANALYSIS

RNA isolation, cDNA synthesis, and semi-quantitative RT-PCR analysis were performed according to Wormuth et al. (2006) using primers as described in **Table 1**. Equal loading of cDNA was adjusted with *ACTIN2* amplificate. Annealing temperatures and amplification cycle numbers were optimized for each target transcript (Oelze et al., 2012).

**Table 1:** Oligonucleotide primers used for (a) cloning of the sAPX promotor fragments into the p35SYFP vector using *Bam*HI and *Nco*I restriction sites and (b) transcript analysis.

	Sequence
**Promoter fragment**
sAPX-1-for	5′-AAAAAGGATCCTTTCGGACCTGGAGAG-3′
sAPX-2-for	5′-AAAAAGGATCCGCACGTCTAGTGAAAGATCC-3′
sAPX-2-mut-for	5′-AAAAAGGATCCGAAAATCTAGTGAAAGATCC-3′
sAPX-3-for	5′-AAAAAGGATCCTGTCAACCAAGTCGCCTTG-3′
sAPX-5-for	5′-AAAAAGGATCCCCCGTCACCATTACCATC-3′
sAPX-6-for	5′-AAAAAGGATCCCTCTATGGACTTTATTGG-3′
sAPX-rev	5′-AAAAACCCATGGTTCTGAGGGGTATAATAGTAAT-3′
**Transcript analysis**
ANAC089-for	5′-ATGGACACGAAGGCGGTT-3′
ANAC089-DB-rev	5′-CAATCAGACGGGCTCCCTG-3′
sAPX-for	5′-ATGCTGCTAACGCTGGTCTT-3′
sAPX-rev	5′-CCTAACGTGTGAGCACCAGA-3′
Actin-2-for	5′-TTGGTAGGCCAAGACATCAT-3′
(At3g18780) Actin-2-rev	5′-GGAGCCTCGGTAAGAAGAAC-3′

### GENERATION OF YEAST-ONE-HYBRID LIBRARY AND SCREENING

cDNA synthesis, construction of Y1H library and screening were performed using the Clontech Matchmaker system as described in Klein and Dietz (2010). To achieve a wide coverage of conditionally expressed transcripts, RNA was isolated from a set of differentially stress-treated *A. thaliana* seedlings. The treatments were as follows: (1) Control: 1 h at 120 µmol quanta m^–2^ s^–1^; (2) combined oxidative and high light stress: 1 h at 5 mM H_2_O_2_ and 1140 µmol quanta m^–2^ s^–1^; (3) drought stress and high light: 1 h drought and 1140 µmol quanta m^–2^ s^–1^; (4) heat stress: 1 h at 40°C and 84 µmol quanta m^–2^ s^–1^; (5) cold and darkness: 1 h at 4°C in darkness; (6) cold in light: 1 h at 4°C and 32 µmol quanta m^–2^ s^–1^; (7) UV-illumination: 3 × UV for 10 s each with regeneration for 30 min at 120 µmol quanta m^–2^ s^–1^; (8) dark-light transition: 1 h darkness followed by 30 min 120****µmol quanta m^–2^ s^–1^; (9) high light: 1 h at about 1100 µmol quanta m^–2^ s^–1^; and (10) low salt: 1 h at 5 mM NaCl and 1100 µmol quanta m^–2^ s^–1^. The bait DNA sequence was cloned into the pHis2 vector using the *Sma*I and *Sac*I endonucleases, while the pGADT7-Rec2 was used as the prey vector. Both vectors were cotransformed into yeast strain Y187. Interaction between fusion protein and DNA-sequence was scored on SD medium supplemented with the appropriate amino acids, i.e., SD/-His/-Leu/-Trp. To suppress leaky His3 activity, 3-amino-1,2,4-triazol (3-AT) was added, for the promoter fragments *sapx2-1* and *sapx2-1*_mut_, the 3-AT concentration was adjusted to 15 mM.

### AMPLIFICATION OF *sapx* PROMOTER FRAGMENTS

*sapx* (At4g08390) promoter fragments were amplified by polymerase chain reaction using the primers listed in **Table 1**. Amplification products were separated by agarose gel electrophoresis, eluted and used for cloning. For transient reporter assays they were fused to EYFP as described in Shaikhali et al. (2008). As reference construct, CFP was fused downstream of the p35S-promoter (Shaikhali et al., 2008). Correctness of constructs was confirmed by DNA sequencing (MWG Biotech, Eberswalde or CeBiTec, Bielefeld, Germany).

### RECOMBINANT PRODUCTION OF ANAC089 PROTEIN AND ELECTROPHORETIC MOBILITY SHIFT ASSAY

The *anac089* coding sequence was amplified using forward (ANAC089-for: 5′-AAAAAAGGATCCATGGACACGAAGGCGGTTG-3′) and reverse primers (ANAC089-rev: 5′-AAAAACTCGAGTTCTAGATAAAACAACATTG-3′) and directionally cloned into pET28a vector using *Bam*HI and *Xho*I restriction sites. The vector was transformed into BL21 (DE3) pLysS *Escherichia coli* cells. Following inoculation with preculture the main culture was grown to OD = 0.6, induced with 400 µM isopropyl-β-D-thiogalactopyranoside and further incubated for 4 h. Cells were sedimented, frozen, and thawn, resuspended in lysis buffer (50 mM Na-phosphate buffer, pH 8, 300 mM NaCl, 10 mM imidazole, 1 mg/ml lysozyme), sonified, incubated and cell debris sedimented by centrifugation. The His_6_-tagged ANAC089 protein was purified by Ni-nitrilotriacetic acid chromatography (Qiagen, Hilden, Germany). Following washing of loosely bound proteins, ANAC089 protein was eluted with elution buffer (50 mM Na-phosphate buffer, pH 8, 300 mM NaCl, 250 mM imidazole). Recombinant protein was dialyzed against 40 mM K-phosphate buffer, pH 7, using dialysis tubing with 10 kDa cutoff. Protein was quantified with BioRad reagent (BioRad Laboratories, München, Germany) with bovine serum albumin as standard. An electrophoretic mobility shift assay (EMSA) was performed with the DIG Gel Shift Kit (2nd generation, Roche, Mannheim, Germany): target DNA was the sAPX promoter fragment 2-1 at an amount of 8 ng per lane. As indicated unlabeled competitor DNA was added at 2 µg concentration and ANAC089 protein at 100 ng. An additional label free EMSA was performed as well. Here recombinant ANAC089 protein equivalent to 360 µM concentration was incubated with 25 ng promotor fragment *sapx-2* (or *sapx-2*_mut_) in 20 µl EMSA buffer (100 mM HEPES, pH 7.5, 500 mM KCl, 25% glycerol, 5 mM dithiothreitol) at 22°C for 30 min. The assay mix was subsequently loaded on 4% agarose gels and the DNA separated by electrophoresis at 80 V. DNA was visualized by ethidium bromide staining and documented.

### CONSTRUCTS FOR FLUORESCENCE MICROSCOPY

ANAC089 was fused to fluorescent proteins for various cell imaging experiments. In each case the enhanced variants of CFP and YFP (ECFP, EYFP) were used for the construction of the vectors. In the following, the short versions CFP and YFP are used in the text. In the transactivation analysis of **Figure 7**, mCherry was fused to ANAC089 (Seidel et al., 2010). The constructs were p35S:CFP:ANAC089, p35S:YFP:ANAC089, p35S:ANAC089:CFP, p35S:ANAC089:YFP, p35S:YFP:ANAC089:CFP, and p35S:mCherry:ANAC089. The cloning strategy included the insertion of the *anac089* gene upstream of the fluorophore gene into the p35S:CFP-NOST and the p35S:YFP-NOST vector using the ANAC089-for (5′-AAAAAAGGATCCAATGGACACGAAGGCGGTTG-3′) and ANAC089-rev (5′-AAAAAACCGGTTCTAGATAAAACAACATT-GC-3′) primers for gene amplification. The *anac089* gene was then fused to the vector utilizing the *Bam*HI and *Age*I endonucleases. The p35S:CFP:ANAC089 and p35S:YFP:ANAC089 constructs were created using the ANAC089-for (5′-AAAAAGCGGCCGCATGGACACGAAGGCGGTT-3′) and ANAC089-rev (5′-AAAAAGAATTCTTATTCTAGATAAAACAACA-3′) primers for the gene amplification which was inserted into the vector using the *Not*I and *Eco*RI endonucleases. The generation of the p35S:YFP:ANAC089:CFP construct was performed in two steps. First the *anac089:cfp* hybrid gene was amplified using the p35S:ANAC089:CFP vector as DNA template. The ANAC089-for (5′-AAAAAAGCGGCCGCATGGACACGAAGGCGGTT-3′) and CFP-rev primer (5′-AAAAAGAATTCTTACTTGTACAGCTCGTC-3′) allowed to insert the amplified hybrid gene into the *Not*I and *Eco*RI restriction sites of the p35S:YFP-C vector, resulting in the p35S:YFP:ANAC089:CFP construct. The various p*sAPX*-YFP promoter fragments were generated using the primer combinations from **Table 1**. The deletion fragments were finally cloned into the pEYFP vector. Gos12 was cloned into 35S-CFP-Nost using the oligonucleotides Gos12-*Bam*HI-for (5′-AAAAGGAT-CCAATGACAGAATCGAGTCTGGAT-3′) and Gos12-*Age*I-rev (5′-AAAAACCGGTGATTTTGAGAGCCAGTAGATGAT-3′). Sar1 (Sar1-*Bam*HI-for 5′-AAAAGGATCCAATGTTCCTGGTGGATT-GG-3′; Sar1-*Age*I-rev 5′-TTTTACCGGTCCGTCGATATATTGA-GA-3′), and Clathrin light chain (ClathrinLC-*Bam*HI-for 5′-AAAAGGATCCAATGTCGTCAACCTTGAGC-3′; ClathrinLC-*Age*I-rev 5′-TTTT-ACCGGTCCCAACTTCTCTGTAAC-3′) were cloned into 35S-CFP-NosT and 35S-YFP-NosT using *Bam*HI and *Age*I restriction sites and plasma membrane ATPase AHA1 (AHA1-*Bam*HI-For 5′-AAAAGCGGCCGCATGTCAGGTCTCGAAGAT-3′; AHA1-*Eco*RI-rev 5′-TTTTTGAATTCTACACAGTGTAGTGA-TG-3′) was cloned into 35S-CFP-C and 35S-YFP-C, respectively, using *Not*I and *Eco*RI restriction sites. Emissions of fused fluorescent protein in cotransfection or transactivation experiments were quantified in the appropriate detector channel of the confocal laser scanning microscope as described in Shaikhali et al. (2008).

### REDOX REGULATION OF THE sAPX PROMOTER

Protoplasts were prepared from At7 cell suspension culture of *A. thaliana* (Seidel et al., 2010). Five days after passage to new medium, cells were sedimented, washed in 50 ml 240 mM CaCl_2_, digested and transfected with *sapx*:YFP promoter constructs as described in Seidel et al. (2004). After incubation at 26°C in darkness for 16 h the different batches were adjusted to the final H_2_O_2_ concentration of 5 mM and DTT of 10 mM, respectively, or left as controls and incubated for further 2 h in darkness at 26°C for 16 h. The relative YFP and CFP emission intensities were measured using the confocal laser scanning microscope (Leica SP2 Heidelberg, Germany). Finally, out of those three different samples the YFP/CFP was calculated.

### CELL IMAGING OF SUBCELLULAR ANAC089 LOCALIZATION AND PROCESSING

Protoplasts prepared from the At7 cell suspension culture were transfected either with the construct combination 35S:ANAC089:CFP and 35S:ANAC089:YFP or 35S:CFP:ANAC089 and 35S:YFP:ANAC089. Homodimerization of ANAC089 *in vivo *was analyzed by Förster resonance energy transfer (FRET; Seidel et al., 2005). Fluorescence of single protoplasts was imaged with a confocal laser scanning microscope (Leica SP2, Heidelberg, Germany). Calculation of FRET efficiency was done as described in Seidel et al. (2005).

### BIOINFORMATIC ANALYSIS OF PROMOTER SEQUENCES AND AMINO ACID SEQUENCES

*sapx* promoter sequences were searched for the presence of putative *cis*-elements with the program Matinspector^1^. The promoters of 27416 *A. thaliana* genes with a length of 3000 bp were downloaded from TAIR^2^ and screened for the ATGCACGTC motif allowing for 1 bp mismatch with the program CLC Main Workbench^3^ . Search for transcripts co-expressed with *sAPX* was performed using the online tool offered at http://www.arabidopsis.leeds.ac.uk/act/coexpanalyser.php. Cleavage site prediction of ANAC089 was performed with ProP1.0^4^ and resulted in two hits, a peculiar Arg/Lys-specific site at amino acid position 163 and a second site at position 297 just close to the membrane spanning α-helix based on prediction from mammalian protease processing sites.

## RESULTS

**FIGURE 1:**
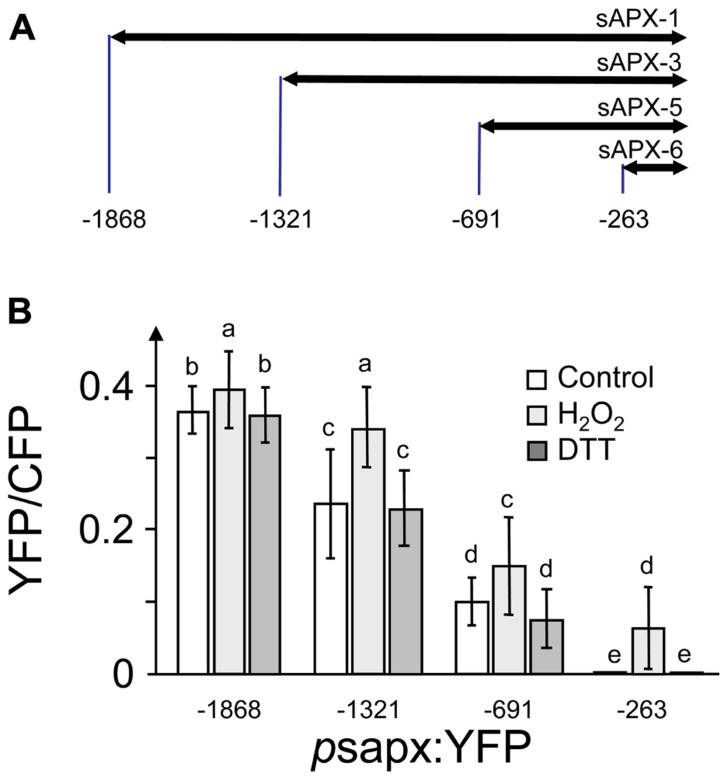
**FIGURE 1. Deletion fragments of the *sapx* promoter constructed for promoter activation assay in *A. thaliana* protoplasts**. **(A)** Schematics of the *sapx* promoter fragments. **(B)** Expression of the YFP reporter gene fused downstream to the *sapx* promoter deletion fragments under control, oxidizing (5 mM H_2_O_2_) and reducing conditions (10 mM DTT) normalized to CFP accumulation under control of the 35S promoter. (1) Promoter fragment p*sapx1*; (2) promoter fragment p*sapx3*; (3) promoter fragment p*sapx5*; and (4) promoter fragment p*sapx6*. Intensities of CFP and YFP were quantified in the respective emission channels of the CLSM (Shaikhali et al., 2008). The results are means ± SD of 60–80 protoplasts. Different letters mark groups of significant difference with *p* < 0.05 (Student’s *t*-test).

The promoter region of *sapx* was cloned as assumed full length or truncated form and fused to enhanced yellow fluorescent protein (YFP) as reporter gene. The selected *sapx1* DNA sequence started from –1868 bp upstream of the translational initiation site down to –1, *sapx3* from –1321 to –1, *sapx5* from –691 to –1 and *sapx6* from –263 to –1. At7-protoplasts were co-transfected with these EYFP reporter constructs simultaneously with a p35S:CFP construct as reference to correct for variable expression levels. The normalized ratios of YFP/CFP were calculated and plotted against the DNA fragment length (**Figure 1**). Transcriptional enhancers were present in all segments since reporter activity decreased with each truncation. H_2_O_2_ stimulated expression driven by each fragment significantly.

**FIGURE 2:**
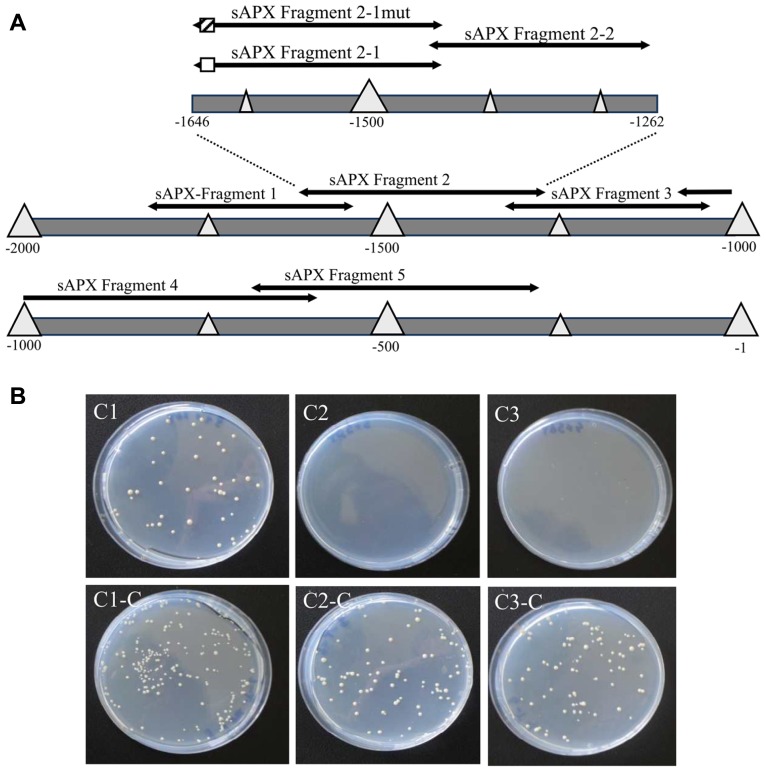
**Schematics of the promoter of *sapx* used for yeast-one-hybrid screening and binding to the specific *cis*-element**. **(A)** Fragment p*sapx1* (–1563 to –1868 bp); p*sapx2* (–1262 to –1646 bp); p*sapx3* (–954 to –1321 bp); p*sapx4* (–609 to –1018 bp); p*sapx5* (–246 to –691 bp); p*sapx6* (–1 to –263 bp). Division of p*sapx2* for localization of the ANAC-binding *cis*-element: pHis2:*sapx2*-1/mut (–1432 bis –1646), pHis2:*sapx2*-2 (–1432 bis –1451). The potential target element for binding of ANAC089 is marked as hatched and the mutagenized region in white box. **(B)** Experimental identification of the binding site of ANAC089 within promoter segment pHis2:*sapx2* using the Y1H system on SD/-Trp/-His/-Leu/+3 AT selective medium (C1, C2, C3) and for control on SD/-Trp/-Leu selective medium (C1-C, C2-C, C3-C). (C1) p His2:*sapx2*-1; (C2) pHis2:*sapx2*-1mut; and (C3) p His2:*sapx2*-2. All cells grew under non-stringent selection showing their viability, while under stringent conditions only the cells grew that were harboring the wild type sapx2-1 fragment and expressing ANAC089.

Since the data of **Figure 1** did not reveal a clear and restricted redox regulation site, the *sapx* promoter was fragmented into five overlapping segments (**Figure 2**) and used for a Y1H screening. 3-AT concentrations to suppress for leaky *His3*-expression without transactivator were adjusted for each construct and were within recommended concentration range for *sapx1* and *sapx2* with 15–20 mM, and already quite high for *sapx3* and *sapx4* (80 and 110 mM, respectively). The *sapx5* fragment remained leaky even at >120 mM 3-AT. This may explain why reliable and specific binding of promising candidates could not be observed for *sapx3* to* 5*. Among the many positive clones only the gene encoding ANAC089 (At5g22290) which was identified as activator of *sapx2* fragment driven reporter expression in yeast appeared promising for further analysis. Retransformation of yeast cells with isolated bait and prey vectors reproduced the interaction between ANAC089 and *sapx2*. Bioinformatic analysis identified two potential binding sites for NAC transcription factors in *sapx2* (sequence position –1646 till –1431, original sequence: GCACGTCTAGTGAAAGATCC, mutated sequence: GAAAATCTAGTGAAAGATCC; second possible binding site was the motive CATCCC at –1627 till –1622). Subcloning confined the transactivation to the *sapx2-1* fragment of 216 bp length (**Figure 2B**, C1) and mutation of the upstream located CACG motif to AAAA (position –1645 to –1642) abolished transactivation of *HIS3* reporter in the *sapx2-1*_mut_:*HIS3* reporter construct after cotransfection with ANAC089 (**Figure 2**, C3).

**FIGURE 3:**
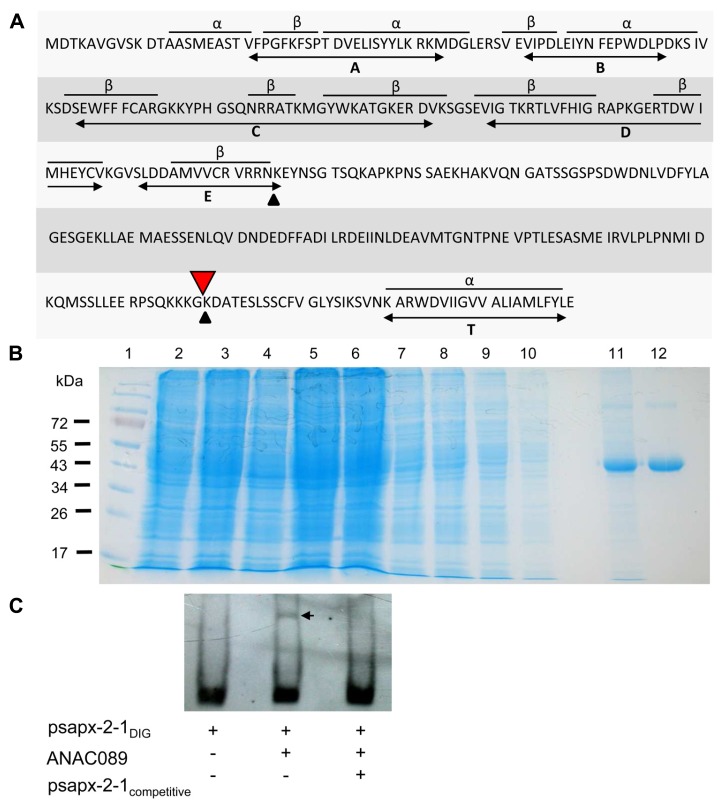
**Structure of ANAC089, generation of recombinant His _**6**_-ANAC089 and EMSA**. **(A)** Primary structure of the ANAC089 transcription factor. The N-terminal ANAC domain consists of three α-helices and eight β-sheets in conserved arrangement (A–E). The bioinformatically predicted transmembrane α-helix (T) of the ANAC089 protein is localized at amino acid position 319–339. Putative protein cleavage sites are indicated with arrows. His_6_-ANAC089 was expressed in *E. coli* and purified by Ni-NTA affinity chromatography. **(B)** Heterologous expression and purification of His6-tagged ANAC089. The recombinant ANAC089 protein was expressed in BL21 (DE3) pLysS *E. coli* cells which were induced with 400 µM isopropyl-β-D-thiogalactopyranoside for 4 h. By SDS PAGE the time-dependent expression of the recombinant ANAC089 protein, as well as the purification process was analyzed. (1) Prestained protein ladder (Fermentas); (2) 0 h after IPTG induction; (3) 2 h after IPTG induction; (4) 4 h after IPTG induction; (5) cell extract; (6) cell pellet; (7–10) column wash fractions; (11) first fraction of ANAC089 elution; and (12) second fraction of ANAC089 elution. **(C)** Electrophoretic mobility shift assay: ANAC089 promoter fragment interaction *in vitro* using the DIG Gel Shift Kit (2nd generation, Roche). Verification of the specificity of ANAC089 binding to the *cis*-element within the sAPX promoter fragment 2-1. Promoter fragment psapx2-1 was loaded at 8 ng DNA. The amount of unlabeled competitor was 2 µg DNA. ANAC089 protein was added at 100 ng. The arrow head marks the shifted band.

Analysis of the *in silico* translated cDNA allowed the identification of the N-terminal NAC domain and a putative C-terminal membrane anchor (**Figure 3A**). ANAC089 protein was recombinantly expressed in *E. coli* as His_6_-tagged protein and purified by Ni-affinity chromatography (**Figure 3B**). Binding of His_6_-ANAC089 to *sapx2-1* promoter fragment *in vitro* was studied using the EMSA (**Figure 3C**). A shift was seen upon addition ANAC089 protein and the shift was abolished upon addition of 250-fold excess competitor DNA. Mutation of the binding site abolished the ANAC089-dependent gel shift (not shown).

**FIGURE 4:**
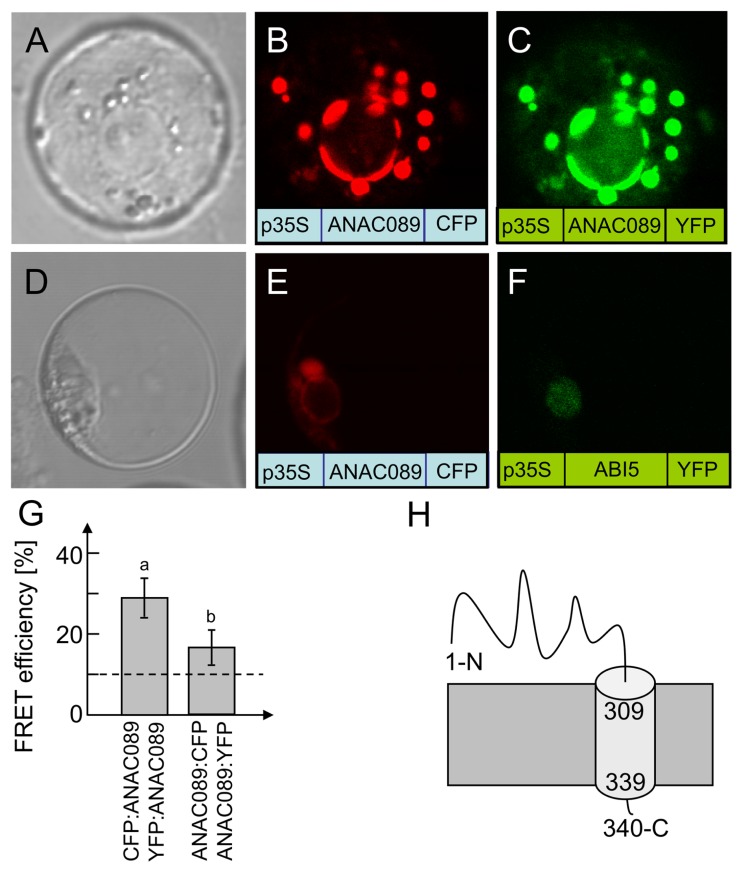
** Subcellular localization of ANAC089 in *A. thaliana* protoplasts**. **(A)** Protoplast in bright field. Fluorescence image of a protoplast expressing the C-terminally CFP-tagged ANAC089 **(B)** or the C-terminally YFP-tagged ANAC089 **(C)**. Comparative localization of ANAC089 und ABI5 with C-terminally fused fluorophores in *A. thaliana *protoplasts. **(D)** Bright field control picture. **(E)** ANAC089-CFP-signal detected in vesicles, endoplasmic reticulum, and plasmamembrane. **(F)** ABI5-YFP-signal in cell nucleus. **(G)** Dimerization detection by FRET analysis: protoplasts were co-transfected with either CFP:ANAC089 and YFP:ANAC089:YFP (left column) or ANAC089:CFP and ANAC089:YFP (right). In both cases significant FRET above the threshold of co-expressed free CFP and YFP could be detected (mean ± SD, *n* > 60 protoplasts, three transfections). **(H)** Putative topology of ANAC089 in the membrane.

At7 protoplasts were transfected with constructs of p35S:ANAC089:CFP and p35S:ANAC089:YFP (**Figure 4**). Both constructs were co-expressed and the reporter fluorescence was observed in the plasma membrane and membrane vesicles (**Figures 4B**,**C**). Analysis for protein–protein interaction by FRET between the CFP- and YFP-fused ANAC089 revealed homooligomer formation (**Figure 4G**). FRET efficiency was significantly above the threshold which was defined as 10% from transfection results with free CFP and YFP (Seidel et al., 2005). FRET was higher if CFP and YFP were fused to the N-terminus of ANAC089. In this case, the fluorophore must have rested in the cytosol. Apparently, also the membrane-associated transcription factor is able to oligomerize. FRET between CFP:ANAC089 and YFP:ANAC089 was at about 28%, whereas the fusion the CFP and YFP fluorophores to the C-terminus of the ANAC089 resulted in 14% FRET efficiency, a value also above the threshold. Lower FRET might be explained by steric constraints, higher distance, or less stable interaction at the C-terminus of ANAC089 and cannot be distinguished by this *in vivo* method.

**FIGURE 5:**
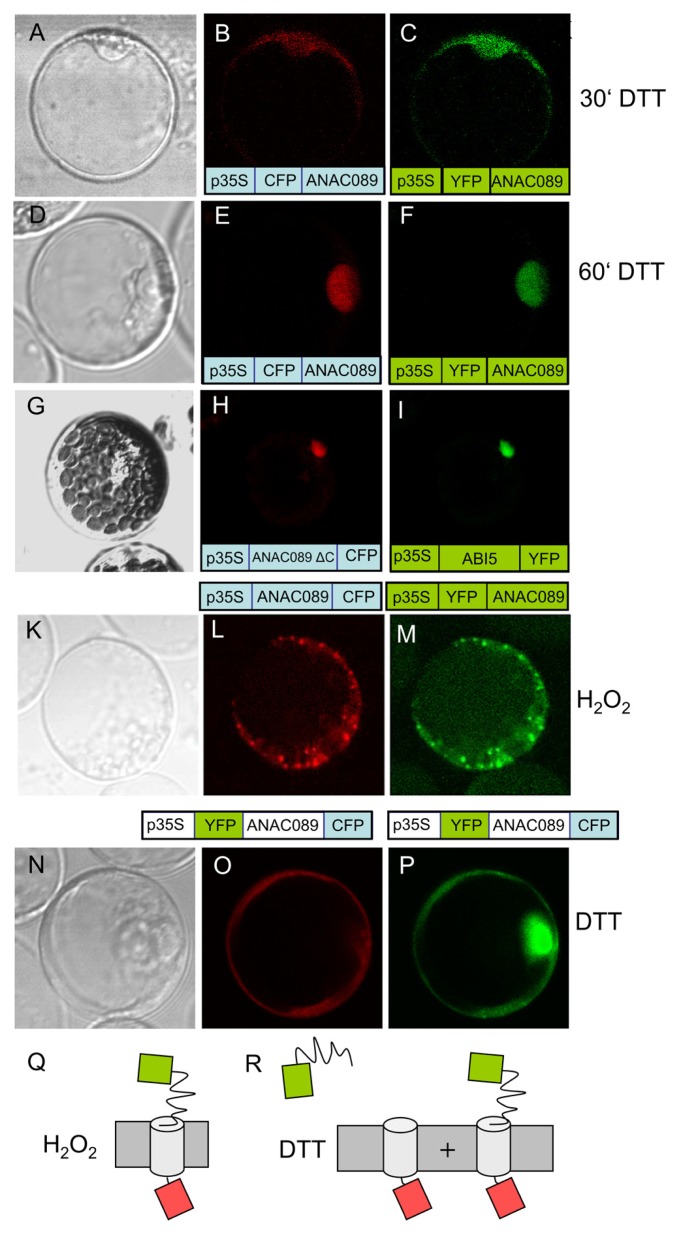
**Redox-dependent dynamics of ANAC089 localization**. **(A**–**F)** Time-dependent localization of ANAC089 was documented following a treatment with dithiothreitol (10 mM DTT). Images were taken at *t* = 30 min **(A**–**C)** and *t* = 60 min **(D**–**F)**. **(A, D)** Bright field image. **(B, E)** Fluorescence signal of N-terminally fused CFP (ANAC089-CFP). **(C, F)** Fluorescence signal of N-terminally fused YFP (ANAC089-YFP). **(G**–**I)** Nuclear accumulation of ANAC089 lacking the transmembrane domain. Protoplasts were transformed with the 3′-truncated ANAC089 coding for a variant that lacks the C-terminal membrane domain (total remaining length 298 aa, see **Figure 3A**, red arrow). In parallel the protoplasts were transformed with ABI5 expression construct. Both fluorophore-fused proteins localized to the nucleus. **(K**–**M)** Localization of N-terminally fused fluorophores in response to H_2_O_2_. Protoplasts of *A. thaliana* were treated with 5 mM H_2_O_2_ for *t* = 60 min. **(K)** Bright field. **(L)** ANAC089:CFP-signal. **(M)** ANAC089:YFP-signal. **(N**–**P)** Translocation of ANAC089 to the nucleus following reductive activation with 10 mM DTT for 60 min. In this case a double fusion was used, namely YFP:ANAC089:CFP under control of the p35S. **(N)** Bright field image. **(O)** CFP signal of the membrane-associated domain. **(P)** YFP signal of the cleaved N-terminal domain of the ANAC089-YFP in the nucleus. The transfection experiments were repeated three times with 70–90% of the successfully transformed protoplasts showing the same result. **(Q,R)** Schematics of underlying model explaining release of YFP to the nucleus following cleavage: CFP in each case rests at the membrane due to its fusion to the membrane-residing helix. The N-terminally fused YFP also rests at the membrane under oxidizing conditions **(M)**, or is partially or fully released with time under reducing conditions which cause cleavage of the ANAC089 domain from the membrane anchor **(P)**.

ABI5, a transcription factor with established localization in the nucleus (Lopez-Molina et al., 2003), was used as a control (**Figure 4F**) and accumulated at a site distinct to ANAC089. Analysis of the coding region with the program TMHMM Server v. 2.0 at http://www.cbs.dtu.dk/services/TMHMM-2.0/ allowed for the identification of a putative transmembrane domain at the C-terminus (**Figure 4H**, see also **Figure 3A**). ANAC089 has been reported before to be a member of a subgroup within the family of NAC transcription factors which is tethered to membranes (Kim et al., 2007). Incubation of transfected protoplasts with dithiothreitol (10 mM) showed time-dependent release of ANAC089 from the membrane and translocation to the nucleus when the fluorophore was fused to the N-terminus of the transcription factor (**Figures 5A**–**F**). ANAC089 lacking the C-terminal membrane domain localized to the nucleus similar to the nuclear transcription factor ABI5 (**Figures 5G**–**I**). ANAC089 remained tethered to the membrane under oxidizing conditions established by addition of 10 mM H_2_O_2_ to the protoplast suspension (**Figures 5K**–**M**). The next experiment addressed the question whether the carboxyterminus rests at the peripheral membranes after cleavage. The protoplasts were transfected with the triple fusion construct YFP:ANAC089:CFP (**Figures 5N**–**P**). Following treatment with DTT, the released YFP:ANAC protein accumulated in the nucleus, while the CFP fused to the carboxyterminus of ANAC stayed at the membrane despite the reductive treatment of the protoplasts.

**FIGURE 6:**
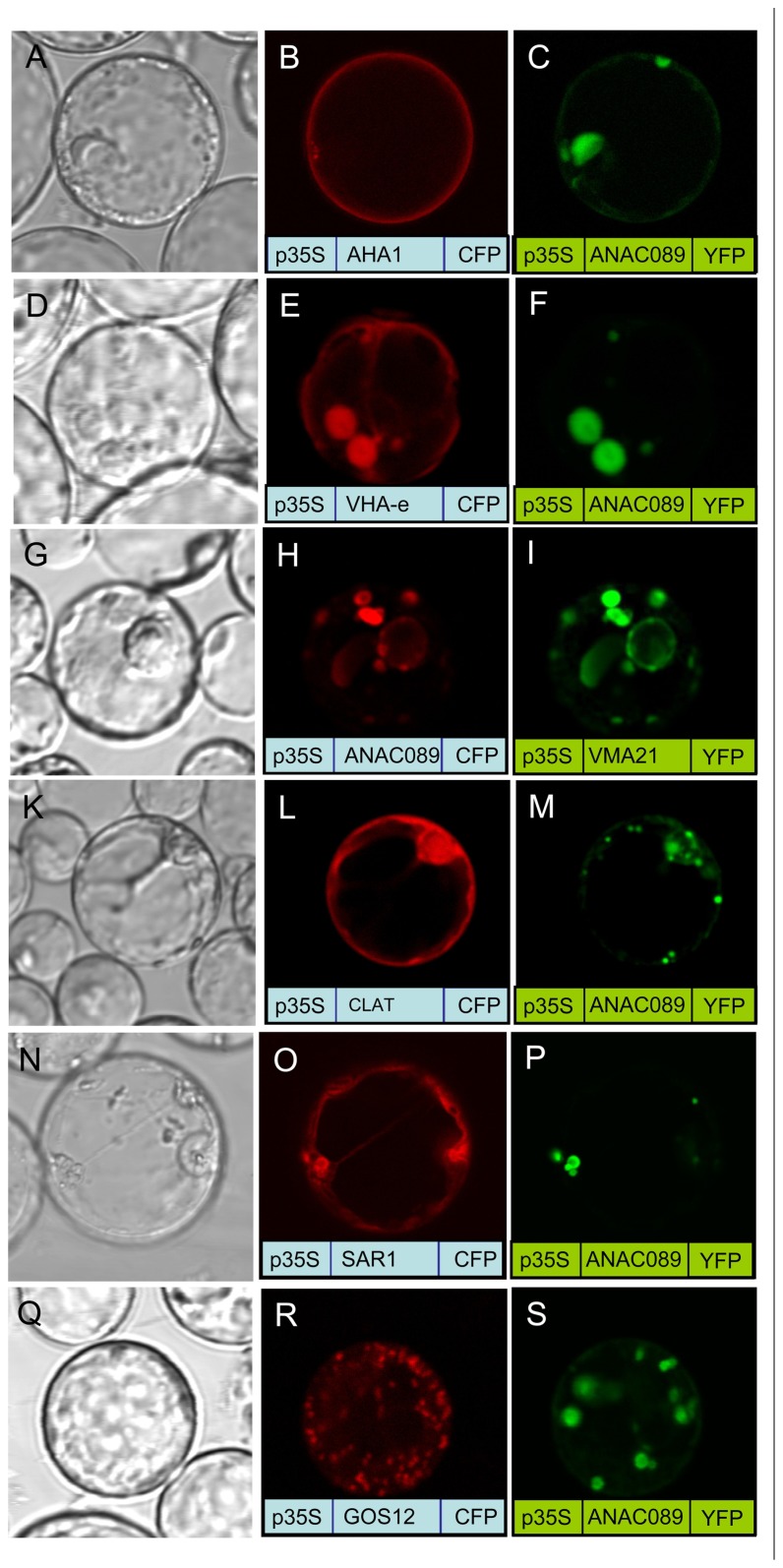
**Co-localization of fluorophore-fused ANAC089 with marker proteins**. Protoplasts were co-transfected with ANAC089 fused to either CFP or YFP as indicated and a marker protein as follows: (**A**–**C)** AHA1, the plasmamembrane H^+^-ATPase, **(D**–**F)** VHA-e, a subunit of the V_0_ domain of V-ATPase that localizes to TGN/EE, **(G**–**I)** VMA21a, an ER-resident assembly factor of the V-ATPas, **(K**–**M)** Clathrin, the light chain of the clathrin coat, **(N**–**P)** Sar1, the small GTPase of COP II vesicle transport anterograde transport between ER and Golgi, and **(Q**–**S)** GOS12, a Golgi SNARE.

The membrane localization of the membrane-anchored ANAC089 was investigated in more detail in protoplasts co-transfected with a set of marker proteins for compartments of the secretory pathway (Uemura et al., 2004; Hanton et al., 2008; Neubert et al., 2008; Seidel et al., 2008; Hwang and Robinson, 2009; **Figure 6**). No co-localization was seen with the plasmamembrane H^+^-ATPase and the Golgi SNARE Gos 12 (**Figures 6A**–**C**,**Q**–**S**), while VHA-e and Vma21a (**Figures 6G**–**I**) showed a high degree of co-localization with ANAC089, respectively (**Figures 6D**–**F**). All other marker proteins, the light chain of Clathrin (CLAT; **Figures 6K**–**M**) and Sar1 (**Figures 6N**–**P**) showed only partial co-localization. The data are consistent with a preferred localization of ANAC089 in the *trans*-Golgi network and in the ER.

**FIGURE 7:**
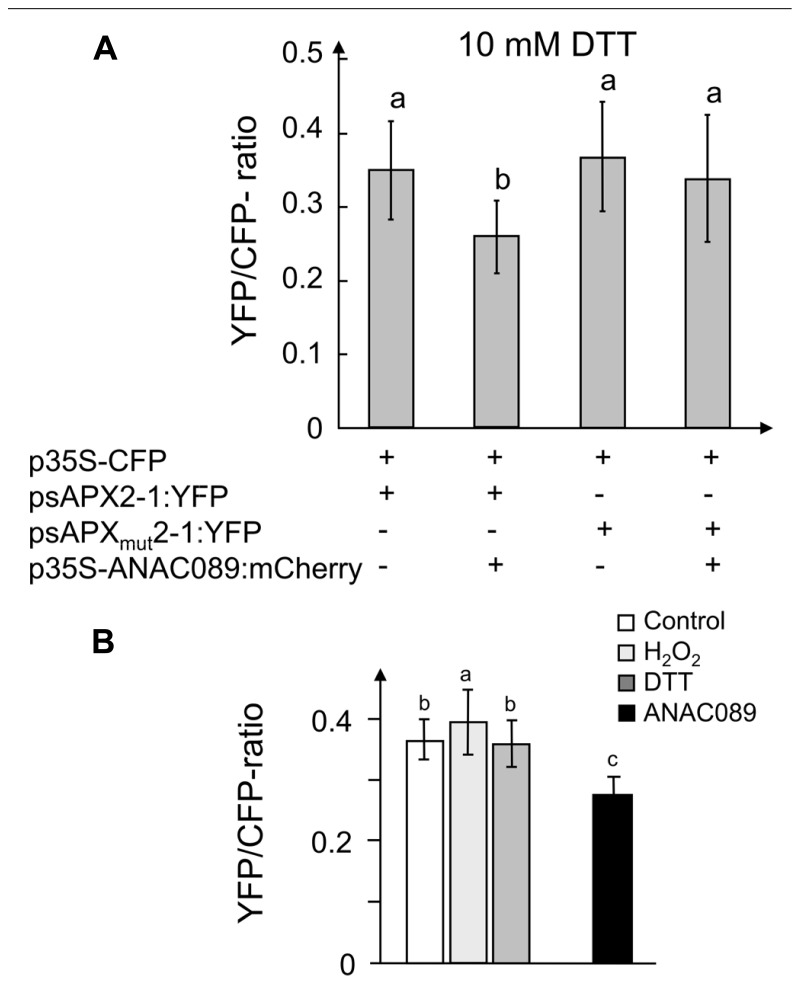
**Effect of overexpressed ANAC089 on reporter gene expression and confirmation of involved *cis*-element CACG**. **(A)** (1st bar) Co-transformation of fragment p*sapx2*-1:YFP with 35S-CFP. (2nd bar) Co-transformation of p*sapx2*-1:YFP with 35S-ANAC089-mCherry and 35S-CFP. (3rd bar) Co-transformation of p*sapx2*-1mut:YFP with 35S-CFP. (4th bar) Co-transformation of p*sapx2*-1mut with 35S-ANAC089-mCherry and 35S-CFP. Prior to the measurement protoplasts were incubated with 10 mM DTT for 2 h. The result represent means ± SD from two experiments with a total *n* > 30 protoplasts per construct. **(B)** A similar effect was seen if the full length promoter (–1 to –1868) was used. The first three columns are as in **Figure1** (expression in control, H_2_O_2_, or DTT-treated protoplasts), the set of three columns on the left hand side represent reporter strength under control conditions, in the presence of H_2_O_2_ and DTT, respectively, the fourth column shows the result with co-expressed p35S:ANAC089. The data are means ± SD from 68, 56, 56, 72, 74, and 83 protoplasts (from left to right column). Different letters indicate significant difference at *p* < 0.05 (Student’s *t*-test).

The regulatory effect of ANAC089 on *sapx* gene expression was studied in transactivation assays (**Figure 7**). *A. thaliana* mesophyll protoplasts were transfected with (i) a reference constructs expressing CFP under the control of the 35S-promoter, (ii) the *yfp* gene placed under control of the *psap2-1* and *psap2-1*_mut_ promoter sequences as transactivation reporter, and (iii) and the construct ANAC089:mCherry as transcriptional modifier. mCherry was fused to the C-terminus of the ANAC089 to check for expression of the *anac089*-gene in transfected protoplasts. The ratio of YFP to CFP was constant in protoplasts transfected with all combinations except for the cells containing the wild type *psap2-1* promoter and expressing ANAC089. Here a significant suppression of YFP reporter intensity by 25% was observed. This suppression was not detected in protoplasts transfected with the mutated promoter proving the importance of the CACG-element in the suppressive regulation. The same suppressive effect was detected when using the full length *sapx* promoter in the absence or presence of co-expressed ANAC089 (**Figure 7B**).

**FIGURE 8:**
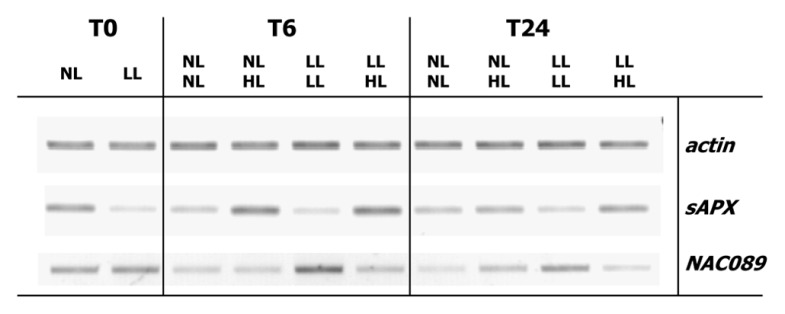
** Regulation of sAPX and ANAC089 transcript amounts in a light shift experiment**. *A. thaliana* were grown for 3 weeks in normal light, either maintained under this condition for another 10 days (80 µmol quanta m^–2^ s^–1^, N-light) or acclimated to very low light (8 µmol quanta^.^m^–2^ s^–1^, L-light) for 10 days. The plants were transferred to high light (800 µmol quanta m^–2^ s^–1^, H-light). Rosettes were harvested at *t* = 0, after 6 or 24 h. RNA was isolated and processed as described by Oelze et al. (2012). Semiquantitative RT-PCR was performed following cDNA synthesis. DNA products were separated by agarose gel electrophoresis and visualized by ethidium bromide staining. Band intensity indicates relative abundance of transcripts.

Finally the relationship between *anac089* and *sapx *gene expression was studied in *A. thaliana* rosettes subjected to a light shift treatment similar to the experiment described in Oelze et al. (2012). Plants fully acclimated to a shady growth condition for 10 days (8 µmol quanta^.^m^–2^ s^–1^) or grown under normal growth conditions (80 µmol quanta^.^m^–2^ s^–1^) were transferred to high light (800 µmol quanta^.^m^–2^ s^–1^) for 6 h. This treatment is known to induce strong regulation of *sAPX* transcript amounts (Oelze et al., 2012). *sAPX* and *ANAC089* transcript amounts were semiquantified and normalized to *ACTIN2* transcript amounts (**Figure 8**). While *sAPX*-mRNA levels were higher in normal light grown plants than in low light acclimated ones, they increased upon transfer to high light. The response was stronger 6 h after transfer than after 24 h. *ANAC089* mRNA levels behaved in a roughly inverse manner. They were high when *sAPX* transcript levels were low and *vice versa*.

In the last step, the presence of the *cis*-element binding ANAC089 in sAPX promoter was searched in the whole *A. thaliana* genome. To this end the frequency of the putative ANAC *cis*-element ATGCACGTC identified by Matinspector with a score of 0.979 was investigated in the whole *A. thaliana* genome. The promoters of 27416 *A. thaliana* genes with a length of 3000 bp were downloaded from TAIR^5^ and screened for the ATGCACGTC motif with the program CLC Main Workbench^6^. The fully conserved ATGCACGTC motif was found in 196 promoters, either in forward or reverse orientation. In 6903 promoters the motif was present with a single nucleotide exchange. *sAPX* co-expression was analyzed at http://www.arabidopsis.leeds.ac.uk/act/coexpanalyser.php. and the 1400 most co-expressed genes were compared with the identified ANAC *cis*-element containing promoters. The obtained list contains candidate genes that might be partially co-regulated with sAPX based on the presence of the ATGCACGTC motif (**Table 2**). Among the genes containing the ATGCACGTC motif (one mismatch allowed) with a co-expression *p*-value > 0.5 are many candidates that show a functional link to stress acclimation and redox metabolism like glutathione reductase, flavonoid biosynthesis enzymes or mitochondrial function.

**Table 2:** Bioinformatic identification of genes that carry the ATGCACGTC motif in their promoter and co-expressed with *sAPX*.

**Identifier**	***p*-value**	***r*-value**	**Function**
AT4G33520	0.628337	8.9e^–37^	Metal-transporting P-type
ATPase AT3G29200	0.611953	1.8e^–34^	Chorismate mutase, chloroplast (CM1)
AT3G55120	0.594613	3.6e^–32^	Chalcone–flavanone isomerase
AT3G24170	0.592559	6.6e^–32^	Glutathione reductase, putative
AT2G45440	0.585632	4.9e^–^^31^	Dihydrodipicolinate synthase 2 (DHDPS2)
AT1G63290	0.569233	4.8e^–^^29^	Ribulose-phosphate 3-epimerase, cytosolic, putative
AT4G16265	0.560068	5.5e^–^^28^	DNA-directed RNA polymerase II, putative
AT1G18320	0.559598	6.3e^–28^	Mitochondrial import inner membrane translocase subunit Tim17
AT3G17609	0.551960	4.5e^–^^27^	bZIP transcription factor/HY5-like protein (HYH)
AT3G06680	0.544932	2.7e^–^^26^	60S ribosomal protein L29 (RPL29B)
AT3G49180	0.530617	8.8e^–^^25^	Transducin family protein/WD-40 repeat family
AT5G44160	0.528320	1.5e^–^^24^	Zinc finger (C2H2 type) family protein
AT2G20450	0.523633	4.5e^–^^24^	60S ribosomal protein L14 (RPL14A)
AT2G26380	0.522760	5.6e^–^^24^	Disease resistance protein-related/LRR protein
AT5G59850	0.519257	1.3e^–^^23^	40S ribosomal protein S15A
AT3G10530	0.519230	1.3e^–^^23^	Transducin family protein/WD-40 repeat family
AT5G20600	0.514334	3.8e^–^^23^	Expressed protein
AT5G50810	0.513390	4.7e^–^^23^	Mitochondrial import inner membrane translocase TIM8
AT5G15910	0.512829	5.4e^–^^23^	NAD(P)-dependent steroid dehydrogenase, related
AT2G23350	0.512790	5.4e^–^^23^	Polyadenylate-binding protein, putative/PABP, putative
AT2G17270	0.510441	9.2e^–^^23^	Mitochondrial substrate carrier family protein
AT4G36020	0.508859	1.3e^–^^22^	Cold-shock DNA-binding family
AT1G60850	0.507399	1.8e^–^^22^	DNA-directed RNA polymerase, putative

*Following a genome-wide search with the ATGCACGTC motif, the hits were compared with the promoters of sAPX-co-regulated transcripts. The list gives the results with a p-value >0.5.*

## DISCUSSION

This work identified and describes the transcription factor ANAC089 as a negative regulator of *sapx* expression (**Figure 7**). The *A. thaliana* genome encodes close to 2,000 putative transcription factors (Iida et al., 2005). The plant-specific NAC transcription factor family comprises about 110 genes in *A. thaliana*. Their usually N-terminally conserved NAC domain of about 60 amino acids was first identified in the three representatives named NAM (no apical meristem), ATAF1/2 and CUC2 (cup shaped cotyledon; Jensen et al., 2010). The NAC domain contains the DNA binding domain, dimerization domain and nuclear localization sequence (Ernst et al., 2004). ANAC089 possesses all these features in its N-terminus.

NAC transcription factors have been shown to be involved in developmental processes, hormonal signaling and responses to abiotic and biotic stresses (Hu et al., 2010). Interestingly, several members of the NAC transcription factor family possess transmembrane anchors. They belong to the membrane-tethered transcription factors (MTTFs). The membrane-associated form is inactive by immobilization. Their proteolytic processing releases the functional TF. The transcript level of *NAC-MTTFs* often is up-regulated under conditions of abiotic stress or in the presence of hormones (Kim et al., 2006). Here it is shown that ANAC089 fusion protein localizes to vesicle-like structures and peripheral membranes. This is in line with the report by Li et al. (2010) showing similar localization. Upon its reductive release from the membrane ANAC089 localized to the nucleus (**Figure 5**). Thus our experiments provide evidence for a signaling cue that triggers its release and gives evidence for process dynamics. The almost exclusive nuclear localization after 60 min in this study was identical to images recorded after expressing a C-terminally truncated variant, ANAC089δC containing amino acids 1 to 310 (Li et al., 2010) and could be confirmed in our cell system after expressing a C-terminally truncated version of ANAC089 lacking the transmembrane domain. Our data indicate that a reductive stimulus is involved in release of the functional TF.

A critical element in the proposed signaling pathway is the protease that releases ANAC089 from the membrane. Conditional proteolysis which is hypothetical at present would allow for rapid transcriptional response to a changing environmental or developmental cue. Up to now, the molecular entities shedding the TFs from their membrane in plants are unknown. In the case of another NAC-MTTF named NTM1, the calpain inhibitor ALLN prevented cleavage from the membrane. Since ALLN specificity is not high, the precise nature of the involved protease remains to be established (Kim et al., 2006). The ANAC089 sequence contains two predicted cleavage sites; a peculiar Arg/Lys-specific site at amino acid position 163 (VVCRVRR|NK) with a high probability and a second site with lower score at position 297 (RPSQKKK|GK) just close to the membrane spanning α-helix based on mammalian protease processing sites (**Figure 3**). About 3% of all *A. thaliana* genes code for proteases (Garcia-Lorenzo et al., 2006) most of which have not been analyzed in any detail. The chloroplast serine Deg1 protease is activated under reductive conditions. It is suggested to localize to the thylakoid membrane and contains many Cys residue, e.g., 10 Cys in *A. thaliana* (Str"o, 2008). Deg proteases function as ATP-independent serine endopeptidases and are found in different organelles and the nucleolus (Deg9) but not in the cytosol or at the endomembranes (Schuhmann and Adamska, 2012). Thus they may serve as examples of principle but unlikely are involved in releasing ANAC089 from their endomembrane attachment site. Three *Arabidopsis* Cys proteases have been shown to be inhibited by binding to protein disulfide isomerase and activated *in vitro *after addition of DTT. The active Cys proteases are involved in cell type-specific programmed cell death in the endothelium of developing seeds (Ondzighi et al., 2008). Thus in addition to the more frequently described oxidative activation of proteases, the given examples reveal that reductive activation of specific proteases as hypothesized here occurs *in planta* as well. The cleavage sites in ANAC089 are predicted for serine proteases of the furin type which are also found in plants. In addition to redox-dependent activation of the protease by disulfide reduction or release from binding partners, a third type of redox regulation might be achieved by burying the cleavage domain of the substrate protein ANAC089 either by conformational changes or posttranslational modifications, e.g., by glutathionylation. ANAC089 contains four Cys residues which may be involved in such a mechanism (**Figure 3A**).

Recently, ANAC089 was linked to sugar sensing in a screen for fructose-insensitive *A. thaliana* mutants. One of the selected mutants *FSQ6* carries an *anac089* allele with premature translational stop in the third exon which enabled growth in the presence of 6.5% (w/v) fructose (Li et al., 2011). This C-terminally truncated ANAC089 in *FSQ6* lacks the membrane spanning helix, thus is not tethered to membranes and immediately translocates to the nucleus upon its synthesis. The expressed ANAC089 variant in *FSQ6* is considered as gain-of function mutation with constitutive ANAC089-dependent target gene expression (Li et al., 2011). Our results demonstrate release of the N-terminus of ANAC089 upon treatment with reductant (**Figure 5**). In their comprehensive search and cataloguing of NAC transcription factors in the rice and *A. thaliana* genomes Ooka et al. (2003) sorted ANAC089 in the group of OsNAC08-related NACs together with ANAC060 and ANAC040. Membrane-tethering of at least 13 ANACs was reported by Kim et al. (2007) the transcript levels of which were often up-regulated under cold, salinity, or heat stress. This contrasts expression of ANAC089. Its mRNA level was high at the end of the dark phase and under low light conditions (**Figure 8**) and down-regulated under high light. High light exposure of normal or low light grown plants imposes excess excitation energy stress and induces a profound transcriptional acclimation response (Khandelwal et al., 2008; Oelze et al., 2012 and unpublished). High light induces oxidative stress and ROS-related signaling (Bechtold et al., 2008). Thus *ANAC089* showed a regulation tentatively reciprocal to the ROS level.

First reports have described cues that lead to the release of membrane-tethered NAC transcription factor. Cytokinin-dependent signaling involves the membrane-anchored ANAC transcription factor NTM1 which regulates cell division (Kim et al., 2006). The MTTF-NAC (At3g49530) mediates part of the cold stress response. Cold temperatures lead to release of the functional NAC-TF from the plasmamembrane over a long time period between 0.5 and 15 h (Seo et al., 2010). In neither of these cases a biochemical cue could be proposed that triggers the shedding. Thus the here reported redox-dependent release offers a first mechanistic interpretation of the processing by intracellular redox signals.

ANAC089 has been shown to be expressed in cotyledons, germinating seedlings, in the vasculature of hypocotyls, roots, rosette and cauline leaves, but also in the interveinal region of the rosette leaves (Li et al., 2010). ANAC089 overexpression delayed flowering time possibly by affecting expression of flower regulatory genes such as CONSTANS (CO) and FLOWERING LOCUS T (FT) whose transcript was suppressed and FLOWERING LOCUS C (FLC) whose transcript accumulation was enhanced (Li et al., 2010). Apparently ANAC089 has multiple functions.

This study assigns an additional and novel role to ANAC089, namely in regulating the expression of sAPX, an important player of the chloroplast antioxidant system. The chloroplast antioxidant system in photosynthesizing cells is expressed at a high level under most conditions (Foyer and Noctor, 2005). This realizes a strong antioxidant defense in a variable environment. However leaves may encounter environmental conditions that prevent photosynthesis-related oxidative stress. Such a reduced state of the glutathione system (Noctor and Foyer, 1998) and the thiol-disulfide redox regulatory network (Dietz, 2008) are likely established if leaves are permanently shaded (Oelze et al., 2012). Then down-regulation of the defense system might save resources for other important synthetic activities. Shedding of ANAC089 from the cytosol-facing side of endomembranes by a thiol redox-regulated process may then allow for translocation of the released functional ANAC089-protein to the nucleus and will subsequently lower *sapx* gene expression. Comparing protein amounts and transcript levels in *A. thaliana* plants acclimated to low, normal, and high light indicates that low levels of *sAPX* transcript are sufficient to maintain high sAPX protein amounts under such conditions probably due to reduced turnover. Such a role of ANAC089 is tentatively supported by the antiparallel transcript regulation of *ANAC089* and *sAPX* in rosettes acclimated and maintained in low and normal light or subsequently transferred to high light (**Figure 8**). According to this scenario, ANAC089 functions in a negative retrograde loop, lowering sAPX expression if the cell encounters highly reducing conditions. In this case the retrograde signal would be a thiol-disulfide redox cue. The putative binding site ATGCACGTC of ANAC089 in the *sapx* promoter is present in many genes tentatively co-expressed with *sAPX*. It will be interesting to investigate the effect of ANAC089 on the expression of these candidate targets including *sapx* by use of transgenic *A. thaliana* lacking or overexpressing ANAC089.

## Conflict of Interest Statement

The authors declare that the research was conducted in the absence of any commercial or financial relationships that could be construed as a potential conflict of interest.

## Footnotes

1 http://www.genomatix.de

2 ftp://ftp.arabidopsis.org

3 http://www.clcbio.com/

4 http://www.cbs.dtu.dk/services/ProP/

5 ftp://ftp.arabidopsis.org

6 http://www.clcbio.com/

## Abbreviations

3-AT: 3-amino-1,2,4-triazol
DTT: dithiothreitol
CFP: (enhanced) cyan fluorescent protein
EMSA: electrophoretic mobility shift assay
FRET: Förster/fluorescence resonance energy transfer
MTTF: membrane-tethered transcription factors
ROS: reactive oxygen species
sAPX: stromal ascorbate peroxidase
SOD: superoxide dismutase
TF: transcription factor
YFP: (enhanced) yellow fluorescent protein.
